# Ovarian Fibrosarcoma: Clinicopathologic Considerations about the Intraoperative and Post-Surgical Procedures

**DOI:** 10.1155/2009/802817

**Published:** 2010-02-07

**Authors:** Angel García Jiménez, Josep Castellví, Assumpció Pérez Benavente, Isabela Díaz de Corcuera Frutos, Santiago Ramón y Cajal

**Affiliations:** ^1^Department of Pathology, University Hospital Vall d'Hebron, Passeig Vall d'Hebron, 119-129, 08035 Barcelona, Spain; ^2^Service of Gynecology, University Hospital Vall d'Hebron, Passeig Vall d'Hebron, 119-129, 08035 Barcelona, Spain; ^3^Service of Medical Oncology, University Hospital Vall d'Hebron, Passeig Vall d'Hebron, 119-129, 08035 Barcelona, Spain

## Abstract

Primary ovarian fibrosarcomas are very uncommon neoplasms. Since the diagnostic criteria were established in 1981, less than one hundred cases have been reported. This diagnosis can be difficult to establish and other similar appearing mesenchymal processes must be ruled out. In every case this diagnosis is under consideration. Multiple sections of the specimen and immunohistochemical stains will be necessary to support this diagnosis. The difficulty of recognition in frozen section in the majority of the situations implies that the diagnosis should be deferred to the definitive study of the permanent sections with immunohistochemical studies. There exists a histological resemblance between a primary ovarian fibrosarcoma and actively mitotic fibroma. In some cases, it can be impossible to separate exactly these two entities. We report a well-differentiated ovarian fibrosarcoma, with less than 1-2 mitosis ×10 HPF and low-grade cytological atypia, similar to active mitotic fibromas, developing liver metastasis one year later. Despite having distant metastasis, some cases with long survival rates have been reported in patients who received chemotherapy after surgery; so that the adjuvant chemotherapy should be considered, especially in young females.

## 1. Introduction

In Gynecology there is a wide spectrum of ovarian spindle cell lesions developing from stromal fibroblasts located at the periphery of ovarian follicles. Amongst these lesions there are fibromas, thecomas, mitotically active fibromas, and fibrosarcomas. The primary ovarian fibrosarcomas are extraordinarily rare neoplasm; the diagnostic criteria include a mitotic index higher than 4 × 10 HPF and atypical cytology. Necrosis and haemorrhage are frequently associated in these cases. Because they are uncommon and can look like other malignant spindle cell processes, the criteria of differentiation sometimes are not clear, especially between mitotically active fibromas and fibrosarcoma. Therefore these lesions may be prone for misdiagnosis leading to in appropriate therapy especially if intraoperative studies with frozen sections are used primarily.

## 2. Clinic Case

**Figure 1:**
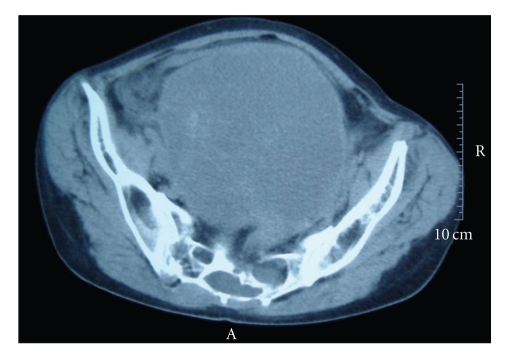
CT scanner image. Huge ovarian mass involving the anterior abdominal wall can be appreciated.

A 55-year-old woman, without significant pathologic antecedents, had vaginal bleeding associated with notable pelvic swelling. A giant heterogeneous ovarian mass of 23 × 15 cm, without lymphadenopathy, was discovered by ultrasound and CT scan image studies (Figure 1). The patient underwent surgical excision of the mass. Ovarian tumour removal was extremely difficult due to the vast adhesions to the anterior abdominal wall as consequence of infiltrative margins. The uterine annexal tumour was sent to the Department of Pathology with the aim to analyze and rule out malignancy by means visual assessment of frozen sections during intraoperative study. The lesion was considered as a spindle cell tumour with no clear signs of atypia, and with final diagnoses deferred to histopathological examination of the permanent sections. Because there were grossly extensive necrotic areas, capsular disruption and many adhesions with other organs, excision using an ovarian cancer protocol was performed. As a result, surgery was completed with bilateral iliac and paraaortic lymph node dissection, omentectomy, and cytological analysis of peritoneal washings.

## 3. Pathologic Study

**Figure 2:**
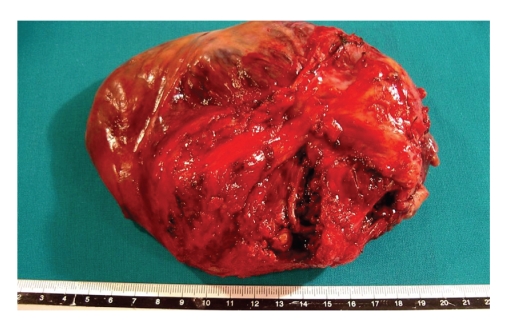
Giant ovarian tumour with capsular disruption and strong red surface colour due to haemorrhaging.

**Figure 3:**
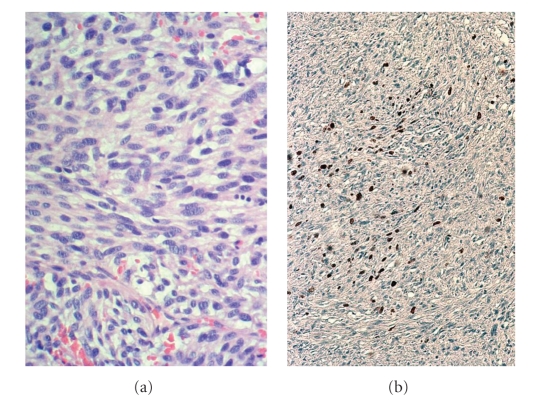
(a) Spindle cells appear with relatively homogeneous nuclei and scant cytoplasm. Low-grade atypia and few number of mitosis can be observed. H/E 400X. (b) the ki-67 immunostain shows a 60% nuclear expression of all spindle cells among the interstitial collagen.

Grossly, the ovarian tumour measured 23 × 15 cm, with capsular disruption and a strong red external surface colour (Figure 2). The cut surface of ovary showed multiple blood filled cysts and solid but necrotic areas. Only 10%–20% of the total of tumour consisted of viable tumour. The specimen was sampled exhaustively. Microscopically, necrosis and haemorrhage were reflected as major components. A proliferation of spindle cells was found, frequently adopting a storiform pattern. The cellular appearance consisted of homogeneously appearing spindle-shaped cells and nuclei with scant amounts of cytoplasm and ill-defined margins (Figure 3). Only a few of mitotic figures was found, with a rate less than 1-2 × 10 HPF. Even though the viable tumour was mainly homogeneous with low number of mitotic figures, the diagnosis of malignancy was rendered due to other factors, such as necrosis, haemorrhage, ovarian capsular disruption, and invasion of the neighbouring tissues. Immunohistochemical stains only revealed a strong expression of vimentin. In order to rule out other mesenchymal entities (GIST, leiomyosarcoma, dendritic cell proliferations, and sarcomatoid carcinomas) other immunostains were performed, such as cocktails of cytokeratins, CD117 (c-kit), smooth muscle actin, H-caldesmon, neuron specific enolase, CD21, CD22, and CN4A. All of these antibofies stained negative in the tumour cells. On the other hand, a nuclear expression for Ki 67 (MIB-1) was seen in more than 60% of total cells, despite the infrequent number of mitotic figures. In the complementary specimens (uterus, lymph nodes, omentum, peritoneum) there was no definitive evidence of malignancy. The case was diagnosed as ovarian fibrosarcoma, stage pT1c No Mo/I C (FIGO). 

After the diagnosis, the patient underwent adjuvant chemotherapy. A combination of 4′-epidoxorubicin 60 mg/m^2^ on days 1 and 2 and ifosfamide 1.8 g/m^2^ on days 1 through 5, with hydration, mesna, and granulocyte colony-stimulating factor were administrated, according to the Italian co-operative study with adjuvant chemotherapy for soft tissue sarcoma [1]. After a disease free interval of 14 months, one metastatic liver nodule was detected. The hepatic lesion was removed and the histology was identical to the primary ovarian tumour. 

## 4. Discussion

Primary ovarian fibrosarcoma is an extremely rare entity [2, 3]. They are considered to have originated directly from stromal cells around the sex cord of ovarian follicles. Alternatively, a malignant transformation of a previous fibroma is also believed to be a potential origin as well. These tumours occur at any age although most of them appear in menopausal and postmenopausal women. Clinically, the patients note abdominal swelling as a result of large pelvic mass with accelerated growth. The majority of tumours show areas of necrosis and haemorrhage, capsular disruption as well as infiltrative margins that make adhesions with other pelvic organs. Microscopically, similar to fibrosarcomas in other locations, there is a high cellularity often there is a moderate cellular pleomorphism and an average of 4 or more mitotic figures × 10 HPF [3]. In some cases, trisomy 12 or 18 has been reported in these neoplasms [4]. Usually, the prognosis is poor, with early distant metastases, showing resistance to some adjuvant chemotherapy regiments, although recently several cases have been reported with long posttreatment survival rates [5]. 

Diagnosis is difficult because there are not specific patterns of immunohistochemical staining or molecular studies for this sort of neoplasm. Before making a diagnosis of primary ovarian fibrosarcoma, other spindle cell lesions should be ruled out. In this sense, some Krukenberg's tumours have a large component of spindle cells, and in some cases the epithelial cells can be identified only after an extensive sampling [6, 7]. Similarity, sarcomatoid carcinomas might have similar histology as fibrosarcoma [6] and require immunohistochemical stains or ultrastructural study to demonstrate their epithelial nature. Primary ovarian leiomyosarcomas and ovarian invasion from uterine origin might be mistaken as fibrous neoplasms. However, leiomyosarcomas tend to show a higher grade of cellular pleomorphism and immunostains with specific markers for smooth muscle. Sometimes, ovarian sex cord tumours are also associated with a vast spindle cell component, mimicking sarcomatoid lesions. In these cases it is necessary to make an exhaustive sampling to minimize misdiagnosis, and in some cases stain for inhibin, although some stromal cells can be positive as well [8]. 

In the differential diagnosis of these spindle-shaped lesions, we should also consider the possibility of gastrointestinal stromal tumours involving the ovary; in these cases, immunostain for CD117 or molecular studies, evaluating c-kit mutations, would be essential [9, 10]. Other processes that eventually affect the ovary are endometrial stromal sarcomas which must be taken into account, especially those with a spindle shaped cell pattern. These cases will need immunohistochemistry with CD10 to demonstrate their stromal nature [11]. More rarely, some tumours with occasional spindle cell or sarcomatoid pattern can involve the ovary such as dendritic cell neoplasms and melanomas, where immunohistochemical stains will be also necessary [12, 13]. Finally, ovarian metastases of sarcomas originating in other locations should also be ruled out. 

Generally, the spectrum of ovarian fibrous tumours is wide and the diagnostic criteria are not always clear and therefore the management of these patients may not be appropriate. There are no problems distinguishing a fibroma from fibrosarcoma; however, the recognition of a mitotically active fibroma is not clear. Mitotically active fibromas have been defined as spindle cell tumours with low mitotic rate (less than 4 × 10 HPF), without atypical cytology, necrosis, or infiltrative margins. On the contrast, ovarian fibrosarcomas usually have higher mitotic rate and have atypical cytology [3]. Usually, these criteria should not give rise to any problems, but Irving et al. recently reported that the mitotic activity was not the unique criteria of malignancy [9]. These authors studied 75 ovarian fibrosarcomas, including 45 mitotically active fibromas. Ovarian fibrosarcomas frequently show cellular atypia or pleomorphism besides a high mitotic activity. In addition, they considered that fibrosarcomas usually had a short history of accelerated growth and were tumours with adhesions, necrosis, and haemorrhage. In contrast, mitotically active fibromas had no cellular atypia, their gross sizes were smaller and adhesions, necrosis or haemorrhage were absent in their series. However, not all of these histological differences can be discriminatory between a fibrosarcoma and mitotically active fibromas. In our case, despite having low degree of atypical cytology and few numbers of mitotic figures, this tumour metastasized to the liver one year later. A discrepant clue in our case leading to our diagnosis of malignancy was the discrepant Ki-67 staining compared to the visual assessment of mitotic figures. Several different series with several tumours with criteria of fibrosarcomas had a good survival outcome and long unexpected survivals after adjuvant chemotherapy have been reported [5]. These experiences bring us the hypothetical idea that mitotically active fibroma and fibrosarcoma could be the same neoplasm with different degree of evolution. In other words, there still exists an overlapping between several ovarian spindle cell tumours that may complicate the suitable management of these patients. 

In conclusion, these findings imply that we still have no clearly defined morphologic criteria to differentiate these proliferative spindle shaped cell processes. The histology is difficult, and there are no specific immunostains or molecular factors capable of identifying the biological behavior. More studies will be necessary to identify predictive factors of behavior. Moreover, the intraoperative studies with frozen sections, that are useful in epithelial tumours, can be extraordinarily difficult to read, and in this sense, no cases of ovarian fibrosarcoma have been reported yet. We recommend caution in the diagnosis by frozen sections. In these situations, we prefer make the diagnosis only as proliferative spindle cell tumour, without excluding a malignant nature and wait to definitive study of permanent sections from extensive sampling. Under these circumstances, gynecological surgeons need to evaluate other additional parameters intraoperatively, such as the existence of capsular disruption, necrosis, or haemorrhage that allows a greater suspicion of malignancy. In these tumours, not only the histological appearance should prevail but the clinical signs (accelerated growth, adhesions) and direct exam of surgical specimen (necrosis, haemorrhage) have to be evaluated as well. In addition, high rates for antigen of proliferation Ki67 (MIB-1), as in our case (above 60%), could contribute to the diagnosis of malignancy, despite having a low visual mitotic rate.
